# Supplementary material for: An approach for developing a blood-based screening panel for lung cancer based on clonal hematopoietic mutations
Source: PLoS One. 2024 Aug 22;19(8):e0307232. doi: 10.1371/journal.pone.0307232 (PMC11341013; doi:10.1371/journal.pone.0307232)
Supplement: Attachment — Submitted filename: Response to Reviewers plosone 20240528.pdf [file pone.0307232.s002.pdf]

Edward Via College of Osteopathic Medicine
VIRGINIA CAMPUS

June 11, 2024

Francesco Bertolini, MD, PhD
Academic Editor
PLOS ONE

Subject: **Response to Reviewer Comments**

New manuscript title: An approach for developing a blood-based screening panel for lung cancer based on clonal hematopoietic mutations**.**

Dear Dr. Bertolini:

We thank the reviewers for reviewing the manuscript referenced above, and for their thoughtful comments.

Overall, we concur with the reviewers' comments. A detailed point-by-point response to reviewer comments is included below. We have revised the manuscript to address reviewer comments and suggestions, as described below. We believe that the value of the manuscript has improved with these revisions. A revised version of the manuscript with tracked changes has been uploaded to the online submission website.

This work was funded by the VCOM 2023 REAP grant #1038624(RA). The funders had no role in study design, data collection and analysis, decision to publish, or preparation of the manuscript.

Thank you for your consideration and we look forward to your feedback.

Ramu Anandakrishnan
Assistant Professor, Biomedical Sciences
Edward Via College of Osteopathic Medicine, Blacksburg, VA, USA
ramu@vt.edu

Point-by-point response to reviewer comments

Reviewer 1 comment:
"The test devised by the authors could indeed complement ctDNA or other diagnostic tests. I wonder whether the presence of cancer in a patient could generate a peripheral blood mixture composed by a different percentage of cell types. Such different cells could harbour different mutations (or mutation rate) due to clonal hematopoiesis."
Response:
This is an interesting question. Cancer is known to alter the peripheral blood immune cell composition, by triggering the expansion of specific immune cell subtypes. These immune cell subtypes may harbor different mutations, which would then be more abundant in the altered peripheral blood due to clonal hematopoiesis (CH). The CH mutations identified by our algorithm may indeed represent the result of CH expansion of specific mutations in specific immune cell subtypes, rather than the general presence of these mutations in hematopoietic stem and progenitor cells. Further investigation will be required to clarify the relationship. We have added a Discussion of this possibility in the manuscript.

Reviewer 1 comment:
"1) It would be useful to encapsulate the test in a software application (with suitable instructions) and provide the possibility to download/run such applications on new datasets."

Response:
We have packaged the code for predicting the probability of cancer from an input VCF files, with instructions for execution. It is available for download from:
https://sourceforge.net/projects/lung-cancer-screening-panel/

Reviewer 1 comment:
"2) It would be interesting to know if you still get a signal completely separating the studies used for training vs tests (i.e. removing the 25% test set)."

Response:
The accuracy for the 8 independent test cohorts (89.88%) was comparable to the accuracy for the 10 cohorts where 25% of the data was set aside for testing (91.42%). These results are included in the Test Results subsection of the Results section.

Reviewer 2 comment:
"The title suggests that the authors provide a panel of genes that can be used to predict the occurrence of lung cancer. The last sentence of the introduction (line 75) also suggests that a machine learning model is developed in this paper to predict the probability of cancer based on the detection of these mutations. However, the results obtained are in contrast in that the authors write that the model developed here may not represent an optimal lung cancer screening panel (line 340) and in that they describe the work as a starting point that needs further development (line 355). Authors must then be consistent with the results by using a title that clearly indicates that a method is presented in this

manuscript and not an already defined panel. Consistency must be checked carefully throughout the manuscript."

Response:

We have updated the title to state that what we present here is "An approach for developing a blood-based screening panel for lung cancer based on clonal hematopoietic mutations". We have also updated the manuscript throughout to indicate that we present an approach that needs to be further validated and optimized.

Reviewer 2 comment:

"At the methodological level, it is not convincing that different alignment and variant calling approaches are used depending on the dataset used. This approach is risky as it could introduce batch effects that may not be corrected by the methods used by the authors. The authors should align reads and perform variant calling with one standardized approach for all datasets and re-run their method."

Response:

For the GDC datasets we used already aligned read files, whereas for the SRA read files we performed the alignment. However, we used the same alignment protocol for SRA dataset as was used for aligning the GDC datasets. Specifically, we used the Burroghs-Wheeler Aligner (BWA) with the GRCh38 reference genome for performing the alignment of SRA datasets, as was used to align the GDC datasets. The same variant calling protocol was then used for all aligned read files (Mutect2). In summary, the same approaches were used for sequence alignment and variant calling for all datasets. We have expanded the Methods section and updated other sections, to clarify.

Reviewer 2 comment:

"In addition, the authors make a number of functional assumptions (line 356) about the identified mutations (Fig. 4) but they do not validate or show data to support the assumptions made."

Response:

On line 356 we stated that "The mutations selected as predictors for the LR model were based on their potentially pathogenic role." The selection criteria are detailed in the Results section under the subsection "Potentially pathogenic CH mutations in TII cells". The values for the selection criteria for each mutation are listed in Supplementary Table S2. The key word here is "potentially". Although the selection criteria used suggest the possibility that these mutations are pathogenic, we do not claim that that is necessarily so. We only suggest that they warrant further investigation. We have expanded the text around line 356 to clarify.

Reviewer 2 comment:

"Additionally on line 349: 48/98 cannot be considered as "most," since it is less than half."

Response:

Most replaced with Many. Thank you.
